# Exploiting *Glycyrrhiza glabra* L. (Licorice) Flavanones: Licoflavanone’s Impact on Breast Cancer Cell Bioenergetics

**DOI:** 10.3390/ijms25147907

**Published:** 2024-07-19

**Authors:** Luca Frattaruolo, Graziantonio Lauria, Francesca Aiello, Gabriele Carullo, Rosita Curcio, Marco Fiorillo, Giuseppe Campiani, Vincenza Dolce, Anna Rita Cappello

**Affiliations:** 1 Department of Pharmacy, Health and Nutritional Sciences, University of Calabria, Via P. Bucci, 87036 Rende, CS, Italy; 2 Department of Biotechnology, Chemistry and Pharmacy, University of Siena, Via A. Moro 2, 53100 Siena, SI, Italy

**Keywords:** Licoflavanone, licorice, flavanones, breast cancer, anticancer agents, energy metabolism, cancer stem cells

## Abstract

Research on the energy metabolism of cancer cells is becoming a central element in oncology, and in recent decades, it has allowed us to better understand the mechanisms underlying the onset and chemoresistance of oncological pathologies. Mitochondrial bioenergetic processes, in particular, have proven to be fundamental for the survival of tumor stem cells (CSC), a subpopulation of tumor cells responsible for tumor recurrence, the onset of metastasis, and the failure of conventional anticancer therapies. Over the years, numerous natural products, in particular flavonoids, widely distributed in the plant kingdom, have been shown to interfere with tumor bioenergetics, demonstrating promising antitumor effects. Herein, the anticancer potential of Licoflavanone, a flavanone isolated from the leaves of *G. glabra*, was explored for the first time in breast cancer cells. The results obtained highlighted a marked antitumor activity that proved to be greater than that mediated by Glabranin or Pinocembrin, flavanones isolated from the same plant matrix. Furthermore, the investigation of Licoflavanone’s effects on breast cancer energy metabolism highlighted the inhibitory activity of this natural product on tumor bioenergetics, a mechanism that could underlie its ability to reduce tumor proliferation, invasiveness, and stemness.

## 1. Introduction

Nature has always represented an invaluable source of bioactive compounds, used over the years as remedies for various pathological conditions, drawing the attention first of traditional medicine and subsequently of phytotherapy and pharmaceutical research. The latter was also able to optimize the pharmacological features of natural compounds to maximize their therapeutic efficacy in order to support conventional therapeutic strategies.

Licorice is a plant of the Glycyrrhiza genus belonging to the Fabaceae family. This genus includes over 30 species widespread in Eurasia, Australia, and America, but the most widely used is *Glycyrrhiza glabra*. 

Besides their crucial role in the food and confectionery industry, the roots of *G. glabra* are the most utilized component of licorice in traditional medicine worldwide, either independently or in combination with other herbal remedies, to treat several health conditions. They act as laxative, anti-inflammatory, anti-ulcer, antibiotic, anti-arthritic, antiviral, memory stimulant, anti-cholinergic, antitussive, anti-caries, hypolipidemic, anti-mycotic, estrogenic, antioxidant, and anticancer agents [1,2,3].

The biological potential of licorice roots is attributable to the variety of active compounds they contain, including hundreds of different polyphenols, classified as phenolic acids, flavonoids, flavones, chalcones, and isoflavonoids, and saponins, such as glycyrrhizin, which is considered the molecule mainly responsible for the beneficial activities of licorice root, particularly the anti-inflammatory one.

On the other hand, *G. glabra* leaves, considered a waste product for the food industry, have been shown in previous studies to contain several flavonoids and dihydrostilbenes with antioxidant, anti-genotoxic, and anti-inflammatory potential, which are either present in small traces or not at all in the roots [4,5]. Moreover, our recent study allowed us to isolate three different flavonoids from the leaves of *G. glabra*, namely Licoflavanone, Pinocembrin, and Glabranin, and to study their anti-inflammatory potential, bringing out an interesting biological potential, in particular for Licoflavanone, triggered by a modulation of the NF-kB transcription factor, which could be widely exploited in human health [6].

Indeed, pathways associated with inflammatory processes are often dysregulated in neoplastic diseases, favoring the proliferation of tumor cells as well as tumor angiogenesis, invasion and metastasis [7,8,9]. This premise, together with the experimental evidence that numerous flavonoids are able to determine antitumor effects thanks to their ability to modulate the redox state of the tumor cell as well as the programmed cell death processes [10], pave the way for studying the antitumor potential of flavanoids present in licorice leaves.

The objective of this study was to evaluate the antitumor activity of three different flavanones isolated from *G. glabra* leaves, Glabranin, Pinocembrin, and Licoflavanone, with the aim of providing more insights into the anticancer potential of these natural products, as well as to better understand their structure-activity relationship and the contribution of the prenyl substituent in the bioactivity of licorice flavanones. In particular, this study allowed us to evaluate and compare the ability of the three flavanones to inhibit breast cancer cell proliferation as well as modulate the cancer hallmarks associated with tumor aggressiveness and invasiveness. Furthermore, recent research showed that two flavonoids from bergamot fruit, Brutieridin and Melitidin, when combined in an enriched fraction (BMF), exhibit great potential in counteracting cancer stem cell (CSC) propagation of MCF7 breast tumor cells. The BMF was able to inhibit various characteristics of CSC behavior, such as mammosphere formation, ALDH level, mitochondrial respiration, and fatty acid oxidation, as well as several signaling pathways involved in stemness [11]. Based on these findings, this work investigated for the first time the effects of Glabranin, Pinocembrin, and Licoflavanone on cancer stem cells, a subpopulation of tumor cells with increased mitochondrial activity and high metabolic flexibility that promote phenomena such as chemoresistance, metastasis, and tumor recurrence, which are responsible in many cases for the failure of current therapeutic regimens [12].

## 2. Results and Discussion

### 2.1. Antitumoral Potential of Glabranin, Pinocembrin, and Licoflavanone

**Figure 1:**
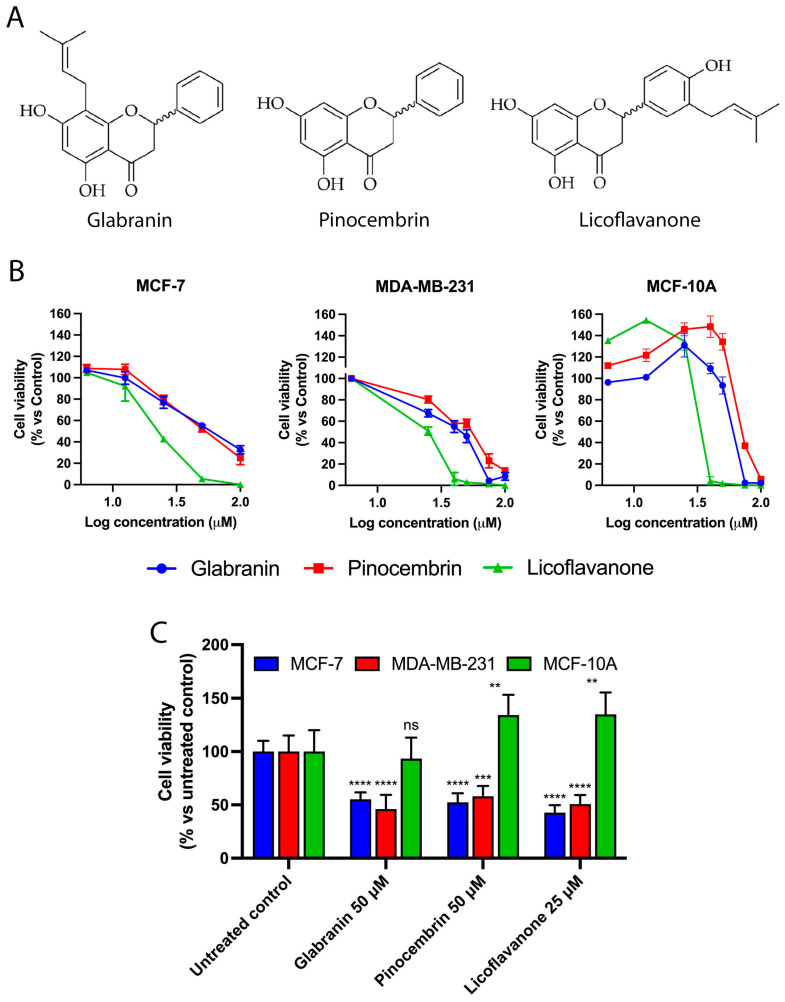
(**A**) Structure of Glabranin, Pinocembrin, and Licoflavanone. (**B**) Cell viability assessment of MCF-7, MDA-MB-231, and MCF-10A cells exposed to different concentrations of Glabranin, Pinocembrin, and Licoflavanone for 72 h. (**C**) Cytotoxic activity comparison between different breast cell lines. Values represent mean ± S.D. of three independent experiments, each performed with triplicate samples. ** *p* < 0.01; *** *p* < 0.001; **** *p* < 0.0001. ns: not significant.

Here, we tested the antitumor potential of Glabranin, Pinocembrin, and Licoflavanone (Figure 1A) in both the luminal breast cancer cell line MCF-7 and the aggressive triple-negative breast cancer cell line MDA-MB-231; the effects of the three flavanones on tumor cell viability were compared to those observed in non-tumorigenic epithelial breast cells MCF-10A. After 72 h of treatment, the results showed the ability of all three compounds to elicit a stronger dose-dependent cytotoxic effect in the two breast cancer cell models than that observed in MCF-10A cells (Figure 1B and Figure S3). Proliferation of tumor and non-tumor cell lines at 24, 48 and 72 h was assessed by MTT assay (Figure S2). The IC_50_ values for each compound were calculated in all cell lines tested (Table 1) and highlighted that MDA-MB-231 cells were the most sensitive to flavanone activity, and, among the three natural compounds, the highest activity was triggered by Licoflavanone. Moreover, our results revealed a fair selectivity of the three different molecules towards the tumor phenotype since, as shown in Figure 1C, 50 µM Glabranin and Pinocembrin as well as 25 µM Licoflavanone drastically reduced tumor cell viability without exerting any cytotoxicity on healthy cells (MCF-10A).

### 2.2. Glabranin, Pinocembrin, and Licoflavanone Induce ROS Accumulation and Apoptosis

**Figure 2:**
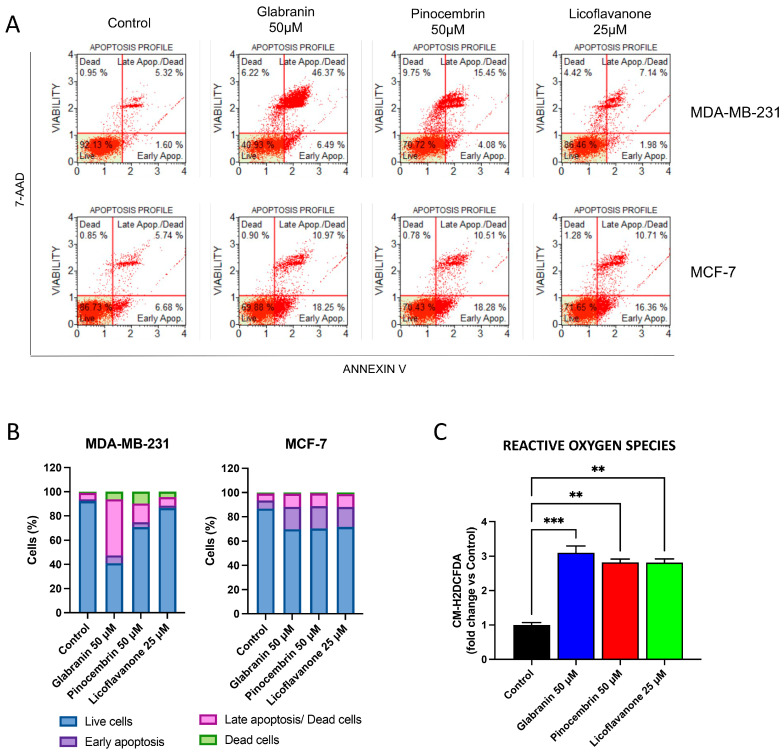
(**A**) Annexin V assay performed in MDA-MB-231 and MCF-7 cells exposed for 48 h to 50 µM Glabranin or Pinocembrin or 25 µM Licoflavanone. (**B**) Quantification of viable, apoptotic, and dead cell populations after 48 h of treatment. (**C**) Reactive oxygen species levels, quantified by using the CM-H2DCFDA fluorescent probe, after MDA-MB-231 cells exposure for 48 h to 50 µM Glabranin or Pinocembrin or 25 µM Licoflavanone. Values represent mean ± S.D. of three independent experiments, each performed with triplicate samples. ** *p* < 0.01; *** *p* < 0.001.

To investigate whether the cytotoxic activity of the three flavanones arises from triggering the apoptotic process, an apoptosis test was performed using the Annexin V assay as reported in the Section 3. The results showed that all three compounds enriched the apoptotic population in both breast cancer cell lines after 48 h of treatment (Figure 2A). A similar apoptotic rate was observed in MCF-7 cells treated with 50 µM Glabranin or Pinocembrin and 25 µM Licoflavanone. On the other hand, in MDA-MB-231 cells, a stronger apoptotic activity was observed after treatment with 50 µM Glabranin or Pinocembrin (Figure 2B). 

Moreover, using the CM-H2DCFDA fluorescent probe, an increase in reactive oxygen species levels was detected after MDA-MB-231 cell exposure to the three different natural compounds (Figure 2C), suggesting that the three flavanones are able to trigger both oxidative stress and apoptosis in breast tumor cells. However, further studies are needed in order to assess additional oxidative stress biomarkers as well as to clarify the molecular pathways linking oxidative stress and apoptotic cell death.

These findings are consistent with the literature data, as the ability of pinocembrin to induce apoptotic death in tumor cells is well documented [13,14,15]; a recent study revealed the ability of Licoflavanone to also induce programmed cell death in nasopharyngeal tumor cells [16]. 

Our results not only corroborate the proapoptotic activity of Licoflavanone in breast cancer cell models, but also, for the first time, allow a comparison between the three different flavanones. This comparison revealed that Licoflavanone is able to elicit the same effects as pinocembrin (its not-prenylated analogue) and Glabranin (its prenylated analogue at a different position) at significantly lower concentrations.

### 2.3. Effect of the Three Different Natural Compounds on Cell Motility of MDA-MB-231 Cells

**Figure 3:**
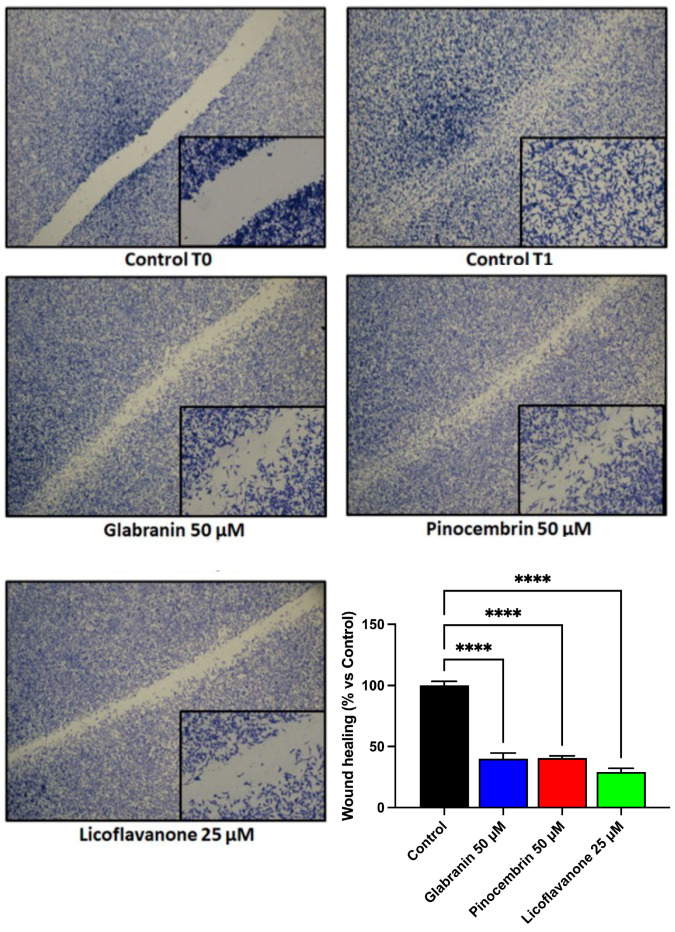
Effects of Glabranin, Pinocembrin, and Licoflavanone on MDA-MB-231 cell motility. Images were taken immediately after wounding (control T0) and after 24 h of treatment, using an Olympus BX41 microscope with CSV1.14 software and a CAMXC-30 for image acquisition. Images were acquired using a 2× objective, while magnifications were obtained with a 20× objective. Histograms indicate % wound healing vs. control. **** *p* < 0.0001.

The ability of cancer cells to detach from the primary tumor and invade distant tissues and organs underlies the metastasizing power of malignant neoplasms. For this reason, to better characterize the antitumor potential of the three flavanones covered in this study, the cell motility of the highly aggressive MDA-MB-231 cell line was monitored by means of the wound healing assay, after 24 h of treatment with 50 µM Glabranin or Pinocembrin or 25 µM Licoflavanone. Untreated cells were used as a control. As shown in Figure 3, the obtained results highlighted the ability of all three compounds to inhibit the motility of these cells. Remarkably, the most pronounced effect was achieved by the 25 µM Licoflavanone treatment, which visibly reduced the “wound healing” more than the treatments with 50 µM Glabranin or Pinocembrin.

### 2.4. Glabranin, Pinocembrin, and Licoflavanone Reduce Macrophage Activation Induced by Breast Cancer-Conditioned Media

Over the past twenty years, inflammation has gained increasing significance in cancer research. It has emerged that some tumors are directly linked to severe inflammatory processes that can promote the development of neoplasms by altering tissue structure [17]. Additionally, interest has grown in the tumor microenvironment as it plays a significant role in tumor progression. Indeed, it is known that cancer cells release various mediators with pro-inflammatory and angiogenic functions, which synergistically cooperate to orchestrate an optimal microenvironment for cancer cell survival and proliferation [18]. 

**Figure 4:**
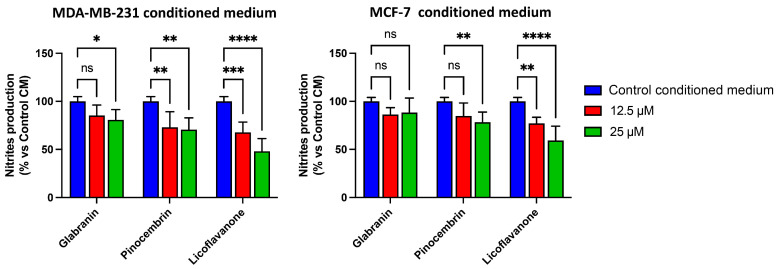
Effects of Glabranin, Pinocembrin, and Licoflavanone on RAW 264.7 macrophage activation induced by breast cancer-conditioned media from MDA-MB-231 or MCF-7 cells (as indicated). Data are the mean ± S.D. of three independent experiments, each performed with triplicate samples. * *p* < 0.05; ** *p* < 0.01; *** *p* < 0.001; **** *p* < 0.0001; ns: not significant.

An important cellular component within the tumor microenvironment consists of tumor-associated macrophages (TAMs), which are known to be activated by cancer cells, thereby releasing biochemical mediators that promote cancer progression and metastatic dissemination. For this reason, TAMs represent an interesting target for potential exploitation in cancer therapy [19,20,21]. Here, we demonstrated the ability of Glabranin, Pinocembrin, and Licoflavanone to modulate RAW 264.7 macrophage activation by MCF-7 and MDA-MB-231 cell-conditioned media, comparing their ability to inhibit the production of nitric oxide (NO), an important inflammatory mediator. Our results demonstrated that NO production was reduced by Pinocembrin and Licoflavanone, while just a slight decrease was observed after Glabranin treatment (Figure 4). Also, in this case, the most noteworthy effect was achieved by the Licoflavanone treatment, which resulted in statistically more significant effects compared to its analogues, Pinocembrin and Glabranin.

It is necessary to underline that, in the experimental conditions used, the three polyphenols did not determine any effect on the viability of macrophages stimulated with conditioned medium. For this reason, the inhibitory effect of the three compounds on NO production cannot be correlated to their cytotoxic activity but rather can be ascribed exclusively to their anti-inflammatory action.

### 2.5. Licoflavanone Modulates the Metabolic Profile of MCF-7 and MDA-MB-231 Cells

**Figure 5:**
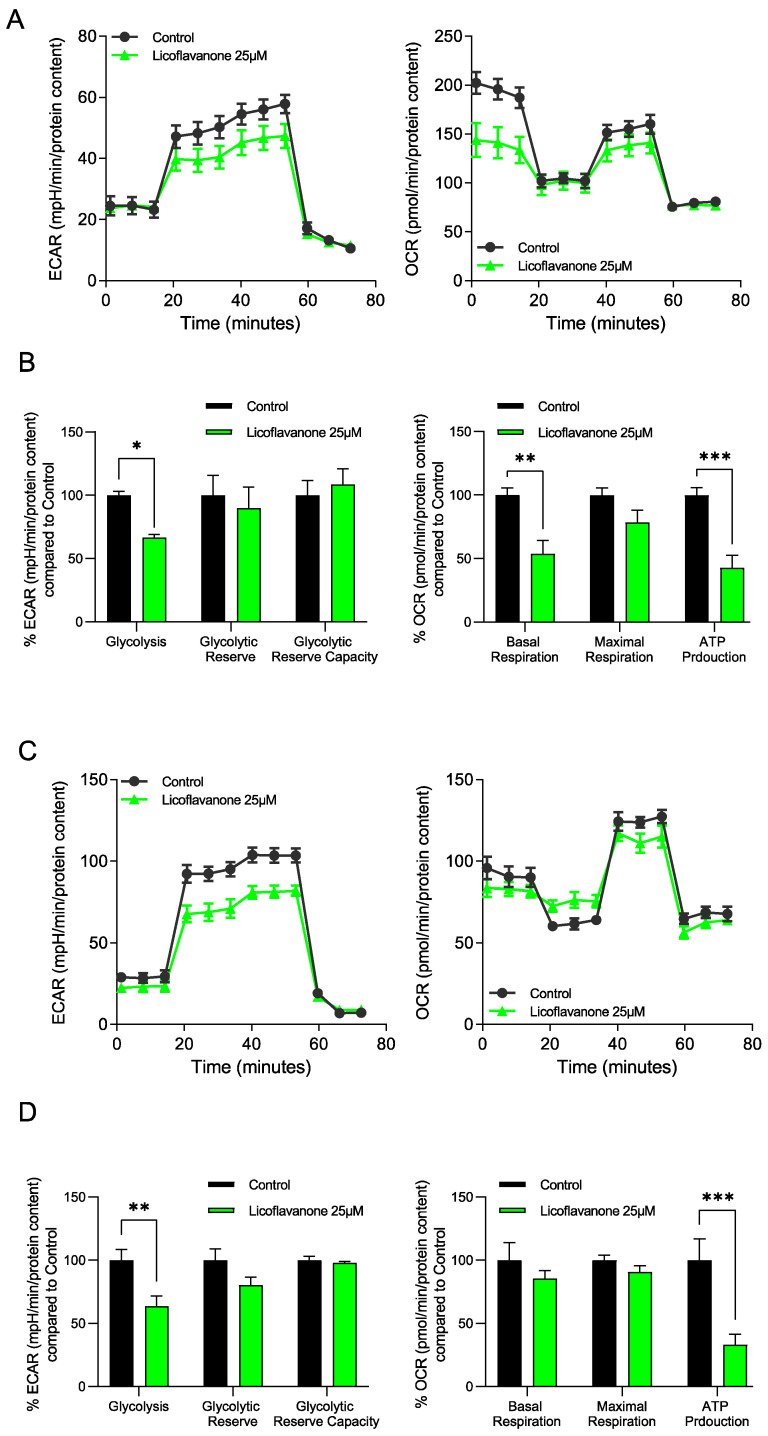
The ECAR (extracellular acidification rate) and the OCR (oxygen consumption rate) were determined using the Seahorse XFe96 via metabolic flux analysis. Note that both MCF-7 (**A**,**B**) and MDA-MB-231 (**C**,**D**) cell populations treated (72 h) with 25 μM Licoflavanone show a decrease in glycolysis as well as a decrease in basal respiration and mitochondrial ATP production compared to control (vehicle-alone) cells. (**A**–**C**) Representative images of the ECAR and OCR were presented. (**B**–**D**) Data represent the % average ± SD over control cells, n = 3. * *p* < 0.05, ** *p* < 0.01, *** *p* < 0.001.

To evaluate if Licoflavanone modulates energy metabolism in cancer cells, the metabolic profiles of both MCF-7 and MDA-MB-231 cells were assessed after 72 h of treatment with 25 μM Licoflavanone. Specifically, using the Seahorse Extracellular Flux (XFe96) analyzer (Agilent, Santa Clara, CA, USA), the glycolytic function of breast cancer cells was evaluated through a Glycolysis Stress test, while the mitochondrial function was assessed by performing a Mito Stress test (as described in the Section 3). The results, depicted in Figure 5, displayed a strong decrease in the oxygen consumption rate linked to ATP production in both cell lines tested when compared to control cells (cells treated with vehicle alone). A significant reduction in basal respiration was also detected in MCF-7 cells. These findings on breast cancer cells treated with Licoflavanone are consistent with the literature, as the inhibitory action of various polyphenolic phytochemicals against the F_O_F_1_-ATPase complex has been widely reported [22,23,24].

### 2.6. Glabranin, Pinocembrin, and Licoflavanone Significantly Inhibits CSC Propagation and Survival by Reducing Stemness Marker Expression

**Figure 6:**
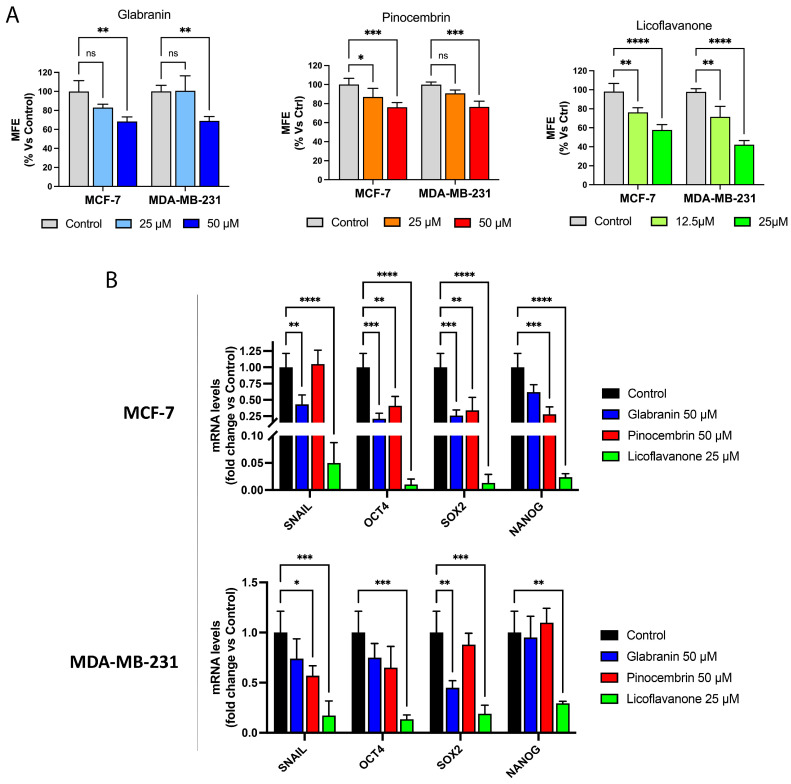
(**A**) Mammosphere formation efficiency (MFE) of MCF-7 and MDA-MB-231 cells treated with 50 µM Glabranin or Pinocembrin or 25 µM Licoflavanone. (**B**) qPCR analysis of mRNA levels of CSC markers (Snail, OCT4, SOX2, NANOG) in MCF-7 and MDA-MB-231 cells exposed to Glabranin, Pinocembrin, and Licoflavanone for 48 h. Values represent mean ± S.D. of three independent experiments, each performed with triplicate samples. * *p* < 0.05; ** *p* < 0.01; *** *p* < 0.001; **** *p* < 0.0001. ns: not significant.

Since Glabranin, Pinocembrin, and Licoflavanone behaved as inhibitors of cell proliferation and motility, we next examined their effects on the behavior of the CSC population, which is believed to be the tumor-initiating cells responsible for chemotherapy resistance, cancer dissemination, and tumor relapse. More specifically, MCF-7 and MDA-MB-231 cells were grown as mammospheres and treated with 25 µM and 50 µM Glabranin, Pinocembrin, and Licoflavanone. Vehicle-alone (control) cells were processed in parallel. Interestingly, Figure 6A shows that all three natural compounds halted mammosphere formation in a dose-dependent manner. In addition, expression levels of validated CSC markers, such as Snail, OCT4, SOX2, and NANOG [25,26,27,28], were determined by qPCR analysis (Figure 6B). 25 µM Licoflavanone diminished the CSC marker expression better than the other two compounds tested, indicating it as a potent inhibitor of CSC formation and propagation. 

### 2.7. Structural Elucidation of Licoflavanone, Glabranin and Pinocembrin

**Figure 7:**
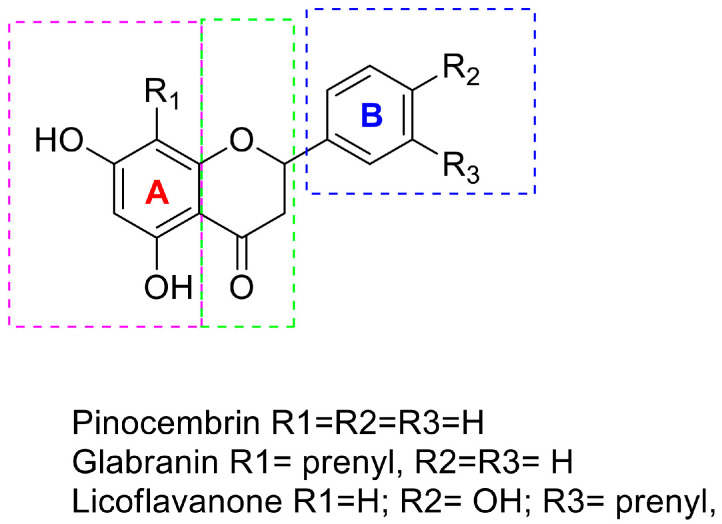
General chemical structure of the assayed compounds.

Pinocembrin, Glabranin, and Licoflavanone share the 5,7-dihydroxy-2-phenylchroman-4-one scaffold (Figure 7). Giving a glance at the in vitro results, the activity of Licoflavanone is always better than that of the other compounds. Lacking a biological target, so far, we have tried to explain this significant difference only using a structure-based approach. The nucleus A is decorated with two hydroxy groups in the C5 and C7 positions, for Pinocembrin and Licoflavanone, with Log *p* = 2.843 and 4.019, respectively, while it shows a prenyl moiety at position C8 in Glabranin with a Log *p* = 4.303 (see Supplementary Material for a complete ADMET profile, Figure S1). The nucleus B in Glabranin and Pinocembrin is free of any substituents. It is well known that the number of hydroxyl groups improves the antioxidant power of all polyphenols, especially when located in nucleus B. In this case, only in Licoflavanone is this condition satisfied by a hydroxyl group in C4′. The contemporary presence of a prenyl chain in C3′ in Licoflavavnone improves the lipophilic behavior with respect to Pinocembrin. Maybe this condition could be responsible for the better activities registered for Licoflavanone. Furthermore, Glabranin seems to be more active than Pinocembrin, from which it differs only in C8 on nucleus A; in this case, the prenyl fragment is the distinguishing element accounting for the different biological profile in the same set of in vitro experiments.

As an example of the capability of a prenyl chain to modulate biological activities, isoflavonoids, a class of secondary metabolites abundant in Leguminosae, can bind to the human estrogen receptor (hER) with affinities similar to or lower than those of estradiol. Dietary intake of these so-called phytoestrogens has been associated with positive effects on menopausal complaints, hormone-related cancers, and osteoporosis. Therefore, phytoestrogens are used as nutraceuticals in functional foods or food supplements. Most of the isoflavonoids show agonistic activity towards both hERα and hERβ, the extent of which is modulated by the substitution pattern of their skeleton (i.e., –OH, –OCH_3_). Interestingly, substitutions consisting of a five-carbon prenyl group often seem to result in antiestrogenic activity. Prenylated flavonoids have been documented to be more bioactive than their flavonoid precursors, as prenylation increases the lipophilicity of flavonoids, leading to higher permeability and bioavailability in vivo [29].

Exploiting mammosphere formation, it is worth noting that dietary supplementation with Piperine and Curcumine is able to modify mammosphere formation [30].

Piperine is a *N*-acylpiperidine that is piperidine substituted by a (1E,3E)-1-(1,3-benzodioxol-5-yl)-5-oxopenta-1,3-dien-5-yl group at the nitrogen atom (PubChem).

**Figure 8:**
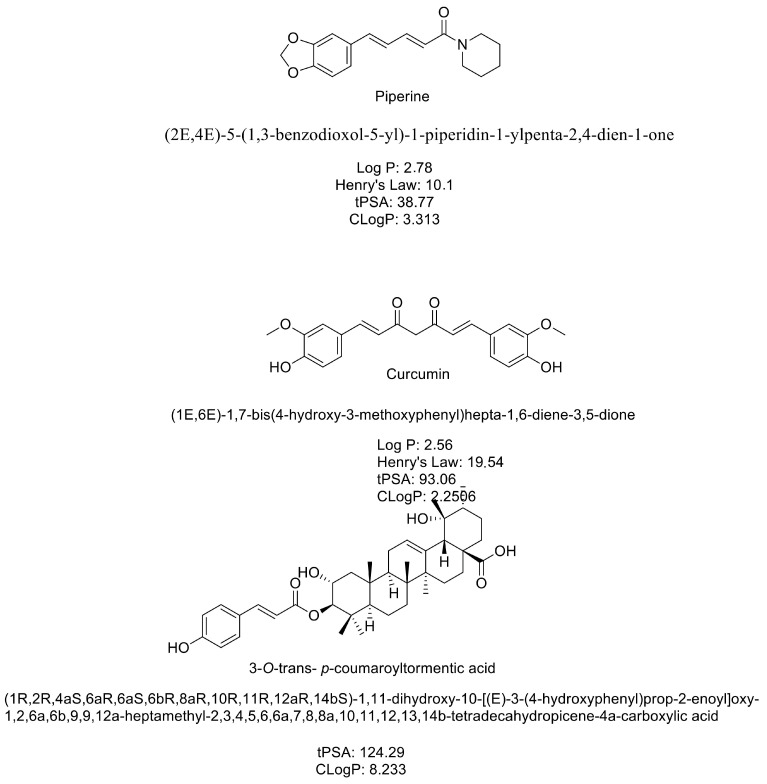
Structure of Piperine, Curcumin, and 3-*O*-trans-*p*-coumaroyltormentic acid.

Curcumin is a beta-diketone that is methane in which two of the hydrogens are substituted by feruloyl groups. Curcumin, also known as diferuloylmethane, is an active component in the golden spice turmeric (*Curcuma longa*) and in Curcuma xanthorrhiza oil. It is a highly pleiotropic molecule that exhibits antibacterial, anti-inflammatory, hypoglycemic, antioxidant, wound-healing, and antimicrobial activities (PubChem). Both of them show an unsaturated chain and hydroxyl group on the phenyl ring, masked as ketals in Piperine (Figure 8).

Triterpene acid (3-*O*-trans-*p*-coumaroyltormentic acid), a triterpenoid isolated from Aronia extracts, effectively inhibits breast cancer cell proliferation and mammosphere formation in a dose-dependent manner. Similarly, several triterpenoids can inhibit cancer stem cells, such as ginsenoside Rg3, a triterpene saponin that inhibits CSCs of colorectal cancer, and gedunin, a natural tetranortriterpenoid that inhibits teratocarcinomal (NTERA-2) cancer stem-like cells. Cucurbitacin E, a tetracyclic triterpene, betulonic acid, a pentacyclic lupane-type triterpenoid, and plant-derived triterpenoid tingenin B inhibit cervical cancer cell lines, leukemia stem cells, and breast CSCs, respectively. Also, triterpene glycosides may be used as adjuvants for the rational design of vaccines against cancer. Phenethyl isothiocyanate, a naturally occurring isothiocyanate, and 8-bromo-7-methoxychrysin, a synthetic derivative of chrysin that is a naturally widely distributed flavonoid, have been shown to possess inhibitory activity against colon and liver CSCs [31].

All these natural compounds are endowed with an unsaturated chain in their scaffold, other than hydroxyl groups.

**Figure 9:**
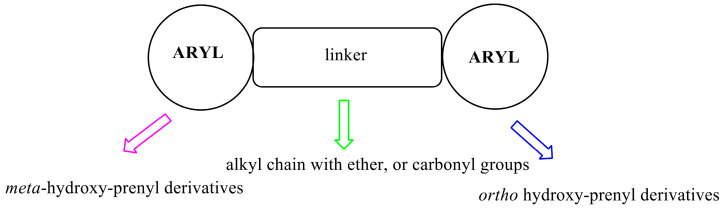
Hypothesis of a suitable lead scaffold.

To better understand the structural relevance of the tested compounds, we split the 5,7-dihydroxy-2-phenylchroman-4-one scaffold into three regions (see Figure 7): nucleus A (purple), nucleus B (blue), and tetrahydro-4*H*-pyran-4-one (green). The green region does not appear to be crucial for biological activity in our in vitro experiments, whereas the blue and purple ones are necessary. Referring to the structure of Glabranin and Licoflavanone, these two portions are, respectively, 4-isopentenyl-1,3-dihydroxybenzene (purple) and 2-hydroxyisopentenyl benzene (blue), and could be considered the pharmacophore. From a medicinal chemistry point of view, applying the scaffold hopping theory, the hypothetical lead scaffold is depicted in Figure 9.

## 3. Materials and Methods

### 3.1. Cell Cultures

Breast cell lines MCF-7, MDA-MB-231, and MCF-10A, as well as murine macrophage RAW 264.7 cell lines, were purchased from the American Culture Collection (ATCC, Manassas, VA, USA). MCF-7 and MDA-MB-231 cells were cultured in DMEM/F12 (Sigma Aldrich, St. Louis, MO, USA) supplemented with 10% Fetal Bovine Serum (FBS), 2 mM l-glutamine, and 1% penicillin/streptomycin (all from Sigma Aldrich). RAW 264.7 cells were cultured in DMEM High Glucose (Sigma Aldrich) supplemented with 10% FBS, 2 mM l-glutamine, and 1% penicillin/streptomycin. MCF-10A cells were cultured in DMEM/F12 supplemented with 5% horse serum (HS), 2 mM l-glutamine, 1% penicillin/streptomycin, 0.5 mg/mL hydrocortisone, 20 ng/mL human epidermal growth factor (hEGF), 10 mg/mL insulin, and 0.1 mg/mL cholera toxin (all from Sigma Aldrich). All cell lines were cultured at 37 °C in 5% CO_2_ in a humidified atmosphere. Licoflavanone, Glabranin, and Pinocembrin were purified as previously described [6]. Treatments were performed in the above-mentioned media containing a lower amount of serum (2%).

### 3.2. Viability Assay

Cell viability was determined by using the 3-(4,5-dimethyl-2-thiazolyl)-2,5-diphenyl-2H-tetrazolium bromide (MTT) assay and the Sulforhodamine B (SRB) assay, as previously described [32,33]. In order to calculate IC_50_ values for each cell line, a non-linear regression analysis (GraphPad Prism 9) was performed to generate sigmoidal dose–response curves.

### 3.3. Apoptosis Assay

MCF-7 and MDA-MB-231 cells were seeded in 6-well plates (2 × 10^5^ cells/well) and treated with different concentrations of compounds for 48 h. They were then subjected to apoptosis assays by means of the Muse^®^ Annexin V & Dead Cell Kit (Merk, Milan, Italy), following the manufacturer’s recommendations. 

### 3.4. Intracellular ROS Assessment

MDA-MB-231 cells were seeded in 6-well plates (2 × 10^5^ cells/well) and treated with the compounds for 24 h. After treatment, the intracellular ROS level in each sample was assessed using the CM-H_2_DCFDA fluorescent dye, as previously reported [33]. Samples were then analyzed by flow cytometry (CytoFLEX Beckman, Beckman Coulter, Milan, Italy). Data analysis was performed using the CytExpert Beckman Coulter software v2.3 (Beckman Coulter, Milan, Italy).

### 3.5. Wound-Healing Scratch Assay

In order to assess the effect of compounds on tumor cell motility, MDA-MB-231 cells were treated for 24 h with 50 µM Glabranin or Pinocembrin and 25 µM Licoflavanone and then subjected to a wound healing scratch assay, as previously reported [34]. 

### 3.6. Nitric Oxide Assessment in RAW 264.7 Cells Stimulated by Tumor-Conditioned Medium

The ability of compounds to modulate the inflammatory process in RAW 264.7 cells exposed to MCF-7 and MDA-MB-231-conditioned media was evaluated by quantifying nitrite levels in macrophages medium by using the Griess reagent (Sigma Aldrich), as previously described [35]. Briefly, breast cancer cells were seeded in 24-well plates at a density of 1 × 10^5^ cells per well and incubated overnight in DMEM/F-12 medium. The following day, in each well, the medium was replaced with a fresh medium for 24 h. Then, RAW 264.7 macrophages were treated with the conditioned media and simultaneously exposed to different concentrations of flavanones (12.5 and 25 µM). After 24 h, the nitrite concentration in each sample was assessed using the Griess assay, following the manufacturer’s recommendations. Basal NO production by unstimulated RAW 264.7 cells was subtracted from the values obtained in stimulated samples.

### 3.7. Metabolic Flux Analysis with the Seahorse XFe96

Real-time extracellular acidification rates (ECARs) and oxygen consumption rates (OCRs) were determined using the Seahorse Extracellular Flux (XFe96) analyzer (Agilent). Briefly, 5 × 10^3^ cells per well were seeded into XFe96-well cell culture plates and incubated for 24 h to allow cell attachment. After 24 h, cells were treated with 25 μM Licoflavanone for 72 h. Vehicle-alone (DMSO) control cells were processed in parallel then washed in pre-warmed XF assay media (or for OCR measurement, XF assay media supplemented with 10 mM glucose, 1 mM Pyruvate, and 2 mM l-glutamine). Cells were then maintained in 175 μL/well of XF assay media at 37 °C in a non-CO_2_ incubator for 1 h. During the incubation time, we loaded 25 μL of 80 mM glucose, 9 μM oligomycin, and 0.5 M 2-deoxyglucose (for ECAR measurement) or 10 μM oligomycin, 10 μM CCCP, 10 μM rotenone, and 10 μM antimycin A (for OCR measurement), in XF assay media into the injection ports in the XFe96 sensor cartridge. Measurements were normalized by protein content (SRB assay). Data sets were analyzed using XFe96 v2.6 software and GraphPad Prism software, using two-way ANOVA and Student’s *t* test calculations. All experiments were performed in quintuplicate, three times independently.

### 3.8. Mammospheres Formation Efficiency

The mammosphere formation efficiency of MCF-7 and MDA-MB-231 cells treated with different concentrations of flavanones (12.5 and 25 µM) was assessed as previously described [36].

### 3.9. qPCR Analysis of Gene Expression

MCF-7 and MDA-MB-231 cells were grown in 10 cm dishes and treated for 48 h with 50 µM of Glabranin or Pinocembrin and 25 µM Licoflavanone. Total RNA was isolated using TRIZOL reagent (Invitrogen, Monza, Italy), following the manufacturer’s procedure. Reverse transcription was performed, as previously reported [37], on each RNA sample to generate complementary DNA (cDNA). Gene expression analyses of SNAIL, OCT4, SOX2, and NANOG were carried out using the Quant Studio 3 Real-Time PCR System (Life Technologies, Monza, Italy) employing the SsoAdvanced Universal SYBR Green (Biorad Laboratories Inc., Milan, Italy), following the manufacturer’s guidelines. Assays were executed in triplicate, and results were normalized using 18S rRNA levels. The ∆∆Ct method was used to calculate the relative mRNA levels. All the oligonucleotides used for qPCR are listed in Table 2.

### 3.10. Statistical Analysis

GraphPad Prism 9 was used to conduct statistical analysis of all the data. Statistical significance was evaluated by an analysis of variance (ANOVA). A *p* value ≤ 0.05 was considered statistically significant. Normality of each distribution was confirmed by the Shapiro–Wilk test, while equality of variance was verified by Levene’s test. Tukey’s post-hoc test was used to study differences between groups.

## 4. Conclusions

In the past decade, the mitochondrial energy metabolism of different tumor cell types has been proposed as a central element for understanding the pathological mechanisms underlying oncological pathology itself and as a possible target for the development of new therapeutic strategies aimed at eradicating it [38,39]. Moreover, mitochondrial metabolism also drives the proliferation of cancer stem cells (CSCs), a subpopulation of tumor cells accountable for tumor recurrence, metastasis onset, and the failure of conventional anticancer therapies [40]. Consequently, investigating molecules capable of influencing mitochondrial metabolism and translating findings into clinical applications could offer an effective approach for treating those tumors strictly dependent on oxidative metabolism [41]. On the other hand, the literature extensively documents that flavonoids, a family of secondary metabolites widely distributed in the plant kingdom and known for their beneficial effects on human health, can exert their actions by modulating various mitochondrial mechanisms [42].

Consistent with the existing literature, our study has uncovered the antiproliferative potential of Licoflavanone from G. glabra leaves, alongside its role in modulating breast cancer aggressiveness and invasiveness. Additionally, our findings have highlighted the heightened potency of this activity compared to Glabranin and Pinocembrin, two flavonoids derived from the same source. Furthermore, this research has shed light, for the first time, on Licoflavanone’s ability to affect mitochondrial metabolism, as demonstrated by the decrease in basal respiration as well as in the oxygen consumption rate linked to ATP production.

These results suggest that the anticancer activity demonstrated by Licoflavanone could be mediated by mitochondrial modulation, as indicated by its ability to inhibit the growth of cancer stem cells, a subset of tumor cells that heavily rely on mitochondrial function for their proliferation.

## Figures and Tables

**Table 1:** Cytotoxic activity of Glabranin, Pinocembrin, and Licoflavanone.

Compound	IC_50_ (µM)		
	**MCF-7**	**MDA-MB-231**	**MCF-10A**
**Glabranin**	68.21	32.50	113.7
	95% CI: 54.09 to 86.81	95% CI: 23.38 to 44.45	95% CI: 61.26 to 247.8
**Pinocembrin**	64.43	48.38	234.5
	95% CI: 48.01 to 87.69	95% CI: 37.09 to 63.02	95% CI: 92.37 to 2637
**Licoflavanone**	19.18	10.97	41.38
	95% CI: 13.21 to 27.34	95% CI: 6.751 to 16.16	95% CI: 19.66 to 88.79

**Table 2:** Sequence of the oligonucleotides used in qPCR analysis of gene expression.

Primer	Forward	Reverse
**SNAIL**	CGAGTGGTTCTTCTGCGCTA	GGGCTGCTGGAAGGTAAACT
**OCT4**	AGCGACTATGCACAACGAGA	CCATAGCCTGGGTACCAAA
**NANOG**	CTCCAACATCCTGAACCTCAGC	CGTCACACCATTGCTATTCTTCG
**SOX2**	GGCCTCGAGCTGGGAATCGC	GCCCACTCGGGGTCTTGCAC

## Data Availability

The original contributions presented in the study are included in the article/Supplementary Materials; further inquiries can be directed to the corresponding authors.

## Supplementary Materials

The following supporting information can be downloaded at: https://www.mdpi.com/article/10.3390/ijms25147907/s1.
