# Supplementary material for: Exploiting Glycyrrhiza glabra L. (Licorice) Flavanones: Licoflavanone’s Impact on Breast Cancer Cell Bioenergetics
Source: Int J Mol Sci. 2024 Jul 19;25(14):7907. doi: 10.3390/ijms25147907 (PMC11276871; doi:10.3390/ijms25147907)
Supplement: Supplementary file 1 [file ijms-25-07907-s001.zip › ijms-3062274-supplementary.pdf]

pkCSM

A

Pharmacokinetic Properties

Glabranin

Molecule Depiction

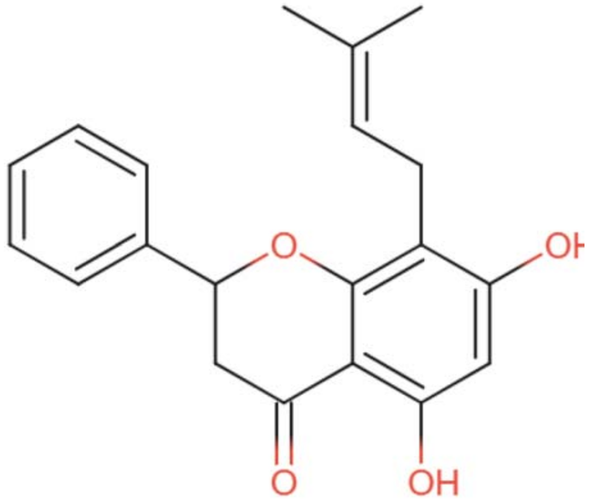

SMILES

Molecule properties:

Descriptor	Value
Molecular Weight	324.376
LogP	4.313
#Rotatable Bonds	3
#Acceptors	4
#Donors	2
Surface Area	140.576

Property	Model Name	Predicted Value	Unit
Absorption	Water solubility	**-3.916**	Numeric (log mol/L)
Absorption	Caco2 permeability	**1.207**	Numeric (log Papp in 10^{-6} cm/s)
Absorption	Intestinal absorption (human)	**92.071**	Numeric (% Absorbed)
Absorption	Skin Permeability	**-2.838**	Numeric (log Kp)
Absorption	P-glycoprotein substrate	**Yes**	Categorical (Yes/No)
Absorption	P-glycoprotein I inhibitor	**No**	Categorical (Yes/No)
Absorption	P-glycoprotein II inhibitor	**No**	Categorical (Yes/No)
Distribution	VDss (human)	**-0.021**	Numeric (log L/kg)
Distribution	Fraction unbound (human)	**0**	Numeric (Fu)
Distribution	BBB permeability	**-0.347**	Numeric (log BB)
Distribution	CNS permeability	**-1.963**	Numeric (log PS)
Metabolism	CYP2D6 substrate	**No**	Categorical (Yes/No)
Metabolism	CYP3A4 substrate	**Yes**	Categorical (Yes/No)
Metabolism	CYP1A2 inhibitior	**Yes**	Categorical (Yes/No)
Metabolism	CYP2C19 inhibitior	**Yes**	Categorical (Yes/No)
Metabolism	CYP2C9 inhibitior	**Yes**	Categorical (Yes/No)
Metabolism	CYP2D6 inhibitior	**No**	Categorical (Yes/No)
Metabolism	CYP3A4 inhibitior	**Yes**	Categorical (Yes/No)
Excretion	Total Clearance	**0.179**	Numeric (log ml/min/kg)
Excretion	Renal OCT2 substrate	**No**	Categorical (Yes/No)
Toxicity	AMES toxicity	**No**	Categorical (Yes/No)
Toxicity	Max. tolerated dose (human)	**-0.153**	Numeric (log mg/kg/day)
Toxicity	hERG I inhibitor	**No**	Categorical (Yes/No)
Toxicity	hERG II inhibitor	**Yes**	Categorical (Yes/No)
Toxicity	Oral Rat Acute Toxicity (LD50)	**1.829**	Numeric (mol/kg)

Property	Model Name	Predicted Value	Unit
Toxicity	Oral Rat Chronic Toxicity (LOAEL)	**1.841**	Numeric (log mg/kg_bw/day)
Toxicity	Hepatotoxicity	**No**	Categorical (Yes/No)
Toxicity	Skin Sensitisation	**No**	Categorical (Yes/No)
Toxicity	*T.Pyriformis* toxicity	**0.759**	Numeric (log ug/L)
Toxicity	Minnow toxicity	**1.17**	Numeric (log mM)

Run another prediction

Back

Instituto René Rachou FIOCRUZ MINAS

THE UNIVERSITY OF MELBOURNE

UNIVERSITY OF CAMBRIDGE

OPEN KNOWLEDGE

Best viewed using Chrome on 1280x1024 resolution and above

B

Pharmacokinetic Properties

Pinocembrin

Molecule Depiction

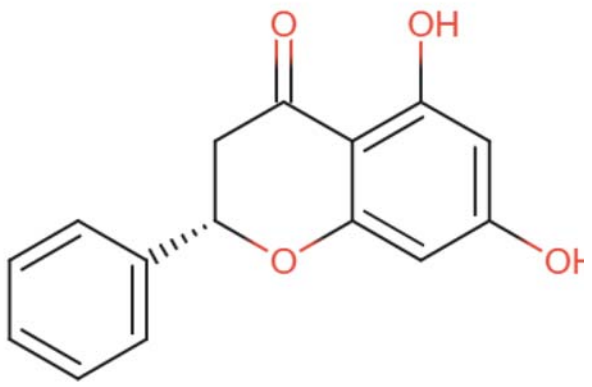

SMILES

Molecule properties:

Descriptor	Value
Molecular Weight	256.257
LogP	2.8043
#Rotatable Bonds	1
#Acceptors	4
#Donors	2
Surface Area	109.441

Property	Model Name	Predicted Value	Unit
Absorption	Water solubility	**-3.31**	Numeric (log mol/L)
Absorption	Caco2 permeability	**1.34**	Numeric (log Papp in 10^{-6} cm/s)
Absorption	Intestinal absorption (human)	**91.223**	Numeric (% Absorbed)
Absorption	Skin Permeability	**-2.879**	Numeric (log Kp)
Absorption	P-glycoprotein substrate	**No**	Categorical (Yes/No)
Absorption	P-glycoprotein I inhibitor	**No**	Categorical (Yes/No)
Absorption	P-glycoprotein II inhibitor	**No**	Categorical (Yes/No)
Distribution	VDss (human)	**-0.255**	Numeric (log L/kg)
Distribution	Fraction unbound (human)	**0.066**	Numeric (Fu)
Distribution	BBB permeability	**0.091**	Numeric (log BB)
Distribution	CNS permeability	**-2.059**	Numeric (log PS)
Metabolism	CYP2D6 substrate	**No**	Categorical (Yes/No)
Metabolism	CYP3A4 substrate	**Yes**	Categorical (Yes/No)
Metabolism	CYP1A2 inhibitior	**Yes**	Categorical (Yes/No)
Metabolism	CYP2C19 inhibitior	**Yes**	Categorical (Yes/No)
Metabolism	CYP2C9 inhibitior	**Yes**	Categorical (Yes/No)
Metabolism	CYP2D6 inhibitior	**Yes**	Categorical (Yes/No)
Metabolism	CYP3A4 inhibitior	**Yes**	Categorical (Yes/No)
Excretion	Total Clearance	**0.216**	Numeric (log ml/min/kg)
Excretion	Renal OCT2 substrate	**No**	Categorical (Yes/No)
Toxicity	AMES toxicity	**No**	Categorical (Yes/No)
Toxicity	Max. tolerated dose (human)	**0.442**	Numeric (log mg/kg/day)
Toxicity	hERG I inhibitor	**No**	Categorical (Yes/No)
Toxicity	hERG II inhibitor	**No**	Categorical (Yes/No)
Toxicity	Oral Rat Acute Toxicity (LD50)	**2.065**	Numeric (mol/kg)

Property	Model Name	Predicted Value	Unit
Toxicity	Oral Rat Chronic Toxicity (LOAEL)	**2.4**	Numeric (log mg/kg_bw/day)
Toxicity	Hepatotoxicity	**No**	Categorical (Yes/No)
Toxicity	Skin Sensitisation	**No**	Categorical (Yes/No)
Toxicity	*T.Pyriformis* toxicity	**0.789**	Numeric (log ug/L)
Toxicity	Minnow toxicity	**0.922**	Numeric (log mM)

Run another prediction

Back

Instituto René Rachou FIOCRUZ MINAS

THE UNIVERSITY OF MELBOURNE

UNIVERSITY OF CAMBRIDGE

OPEN KNOWLEDGE

Best viewed using Chrome on 1280x1024 resolution and above

pkCSM

C

Pharmacokinetic Properties

Licoflavanone

Molecule Depiction

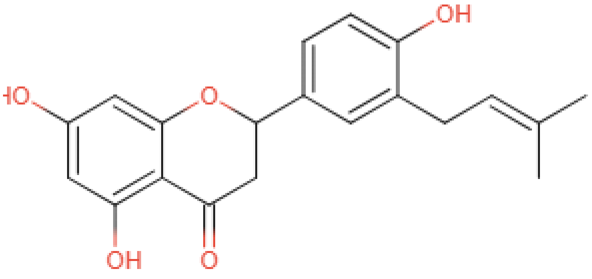

SMILES

Molecule properties:

Descriptor	Value
Molecular Weight	340.375
LogP	4.0186
#Rotatable Bonds	3
#Acceptors	5
#Donors	3
Surface Area	145.370

Property	Model Name	Predicted Value	Unit
Absorption	Water solubility	**-3.75**	Numeric (log mol/L)
Absorption	Caco2 permeability	**1.084**	Numeric (log Papp in 10^{-6} cm/s)
Absorption	Intestinal absorption (human)	**89.406**	Numeric (% Absorbed)
Absorption	Skin Permeability	**-2.821**	Numeric (log Kp)
Absorption	P-glycoprotein substrate	**Yes**	Categorical (Yes/No)
Absorption	P-glycoprotein I inhibitor	**No**	Categorical (Yes/No)
Absorption	P-glycoprotein II inhibitor	**Yes**	Categorical (Yes/No)
Distribution	VDss (human)	**0.163**	Numeric (log L/kg)
Distribution	Fraction unbound (human)	**0.133**	Numeric (Fu)
Distribution	BBB permeability	**-0.965**	Numeric (log BB)
Distribution	CNS permeability	**-2.07**	Numeric (log PS)
Metabolism	CYP2D6 substrate	**No**	Categorical (Yes/No)
Metabolism	CYP3A4 substrate	**No**	Categorical (Yes/No)
Metabolism	CYP1A2 inhibitior	**Yes**	Categorical (Yes/No)
Metabolism	CYP2C19 inhibitior	**Yes**	Categorical (Yes/No)
Metabolism	CYP2C9 inhibitior	**Yes**	Categorical (Yes/No)
Metabolism	CYP2D6 inhibitior	**No**	Categorical (Yes/No)
Metabolism	CYP3A4 inhibitior	**No**	Categorical (Yes/No)
Excretion	Total Clearance	**0.165**	Numeric (log ml/min/kg)
Excretion	Renal OCT2 substrate	**No**	Categorical (Yes/No)
Toxicity	AMES toxicity	**No**	Categorical (Yes/No)
Toxicity	Max. tolerated dose (human)	**0.01**	Numeric (log mg/kg/day)
Toxicity	hERG I inhibitor	**No**	Categorical (Yes/No)
Toxicity	hERG II inhibitor	**Yes**	Categorical (Yes/No)
Toxicity	Oral Rat Acute Toxicity (LD50)	**2.292**	Numeric (mol/kg)

Property	Model Name	Predicted Value	Unit
Toxicity	Oral Rat Chronic Toxicity (LOAEL)	**1.663**	Numeric (log mg/kg_bw/day)
Toxicity	Hepatotoxicity	**No**	Categorical (Yes/No)
Toxicity	Skin Sensitisation	**No**	Categorical (Yes/No)
Toxicity	*T.Pyriformis* toxicity	**0.424**	Numeric (log ug/L)
Toxicity	Minnow toxicity	**1.034**	Numeric (log mM)

Run another prediction

Back

Instituto René Rachou
FIOCRUZ MINAS

THE UNIVERSITY OF MELBOURNE

UNIVERSITY OF CAMBRIDGE

OPEN KNOWLEDGE

Best viewed using Chrome on 1280x1024 resolution and above

Figure S1. Pharmacokinetic properties of (A) Glabranin, (B) Pinocembrin and (C) Licoflavanone

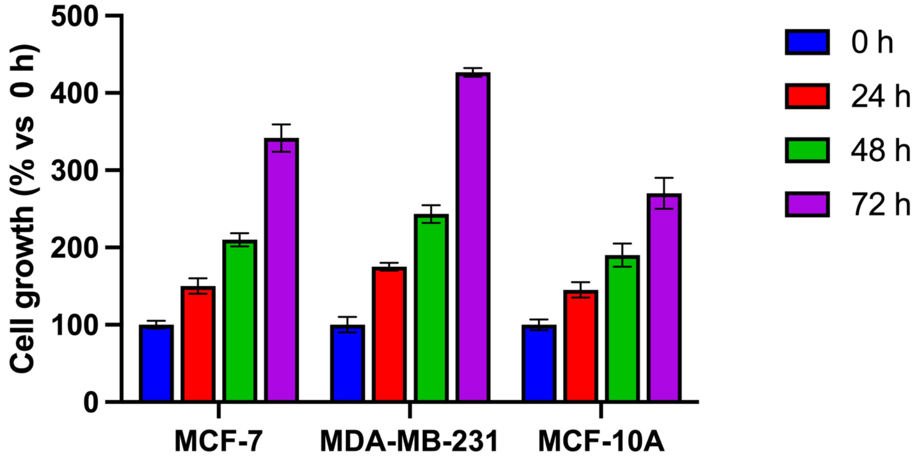

Figure S2. Cell growth of MCF-7, MDA-MB-231 and MCF-10A, assessed by MTT assay.

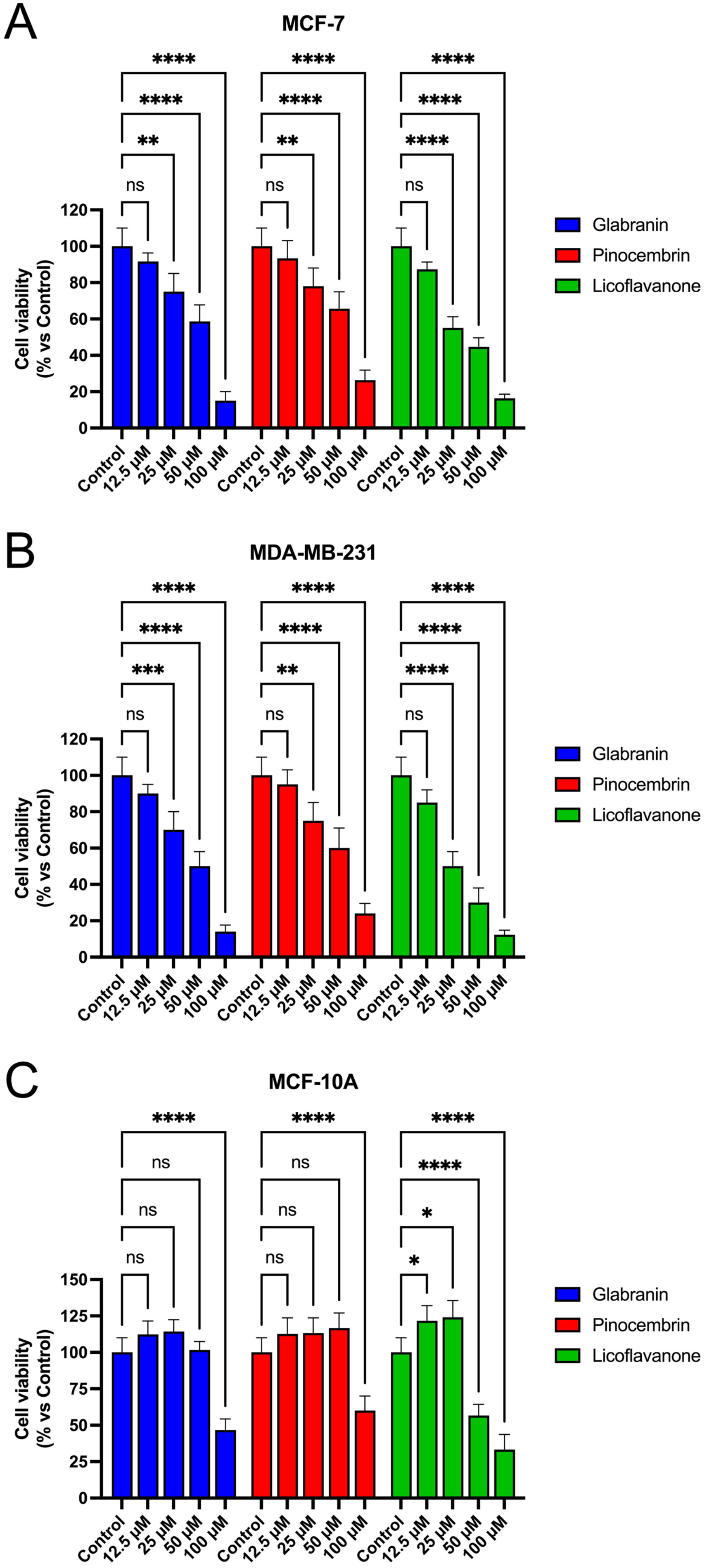

Figure S3. Cell viability assessment, by SRB assay, of MCF-7 (A), MDA-MB-231(B) and MCF-10A (C) exposed to different concentrations of Glabranin, Pinocembrin and Licoflavanone, for 72 hours. Values represent mean ± S.D. of three independent experiments, each one performed with triplicate samples. * $P<0.05$; ** $P<0.01$; *** $P<0.001$; **** $P<0.0001$. ns: not significant.
